# Tissue accumulation of microplastics in mice and biomarker responses suggest widespread health risks of exposure

**DOI:** 10.1038/srep46687

**Published:** 2017-04-24

**Authors:** Yongfeng Deng, Yan Zhang, Bernardo Lemos, Hongqiang Ren

**Affiliations:** 1 State Key Laboratory of Pollution Control and Resource Reuse, School of the Environment, Nanjing University, Nanjing, Jiangsu 210023, China; 2 Program in Molecular and Integrative Physiological Sciences, Department of Environmental Health, Harvard T. H. Chan School of Public Health, Boston, MA 02115, USA

## Abstract

Microplastics (MPs) are a significant environmental health issue and increasingly greater source of concern. MPs have been detected in oceans, rivers, sediments, sewages, soil and even table salts. MPs exposure on marine organisms and humans has been documented, but information about the toxicity of MPs in mammal is limited. Here we used fluorescent and pristine polystyrene microplastics (PS-MPs) particles with two diameters (5 μm and 20 μm) to investigate the tissue distribution, accumulation, and tissue-specific health risk of MPs in mice. Results indicated that MPs accumulated in liver, kidney and gut, with a tissue-accumulation kinetics and distribution pattern that was strongly depended on the MPs particle size. In addition, analyses of multiple biochemical biomarkers and metabolomic profiles suggested that MPs exposure induced disturbance of energy and lipid metabolism as well as oxidative stress. Interestingly, blood biomarkers of neurotoxicity were also altered. Our results uncovered the distribution and accumulation of MPs across mice tissues and revealed significant alteration in several biomarkers that indicate potential toxicity from MPs exposure. Collectively, our data provided new evidence for the adverse consequences of MPs.

In recent years, plastic particles with a diameter <5 mm have been increasingly recognized as a global environmental threat and health hazard to human populations^1^,^2^. These microplastics (MPs) are produced through two main sources: (1) manufactured products contained plastic particles or powders, such as cosmetics, detergents, sunscreens, and drug vectors^3^; (2) breakdown of larger piece of plastics through UV-radiation, mechanical abrasion, and biological degradation in the environment^4^. MPs have been detected in environments and media as varied as oceans, rivers, sediments, sewages, soil and even table salts^5^,^6^,^7^,^8^,^9^. Human populations can be exposed to MPs directly from the environment or through the food. Multiple studies have documented that MPs are ingested by various marine organisms (bivalves, zooplankton, copepods, fish, etc)^10^,^11^,^12^,^13^, accumulate in specific tissues (gill, gut, digestive gland, circulatory system, etc.)^14^,^15^,^16^, and are transported up through the food chains (e.g., transfer in planktonic food web, from mussel to crab, through zooplankton to fish, etc)^17^,^18^,^19^. Hence, widespread use and persistence of MPs is expected to lead to its accumulation in the environment and greater exposure risk for wild organisms and human populations over time.

Previous studies have demonstrated that the accumulation and distribution of MPs in aquatic organisms is species-specific and can be influenced by particle size. For example, 8–10 μm MPs mainly reside in crab gill and gut^14^; 10 μm MPs can be transported into the circulatory of mussels^20^; 5 μm MPs can be accumulated in the liver of zebrafish^21^. While most researches on the toxic effects of MPs have focused on aquatic organisms, studies documenting the potential health risk and tissue accumulation of MPs in mammals are lacking. Tissue accumulation of MPs may induce various adverse effects, such as physical injury^22^, reduction of feeding activity^23^, inhibition of growth and development^24^, energy deficiency^3^, immune responses^25^, oxidative stress^26^, neurotoxic responses^27^, metabolic disorder^28^, and genotoxicity^13^. Hence, data on tissue accumulation of MPs in mammalian models would be indispensable for risk assessment of MPs in human health.

The purpose of this study is to quantify the distribution and accumulation of MPs in mice (*Mus musculus*) tissues based on fluorescence spectroscopy, and address toxicological responses to MPs exposure using enzymatic biomarkers and metabolomic profiles. The results of this study provide new insights about the potential health risk of MPs exposure.

## Methods

### MPs particles

Polystyrene microplastic (PS-MPs) particles were obtained from BaseLine ChromTech Research Centre (Tianjin, China) in a distilled water carrier. Two types of beads were used in this study. One type was fluorescent PS particles with an excitation and emission wavelengths of 418 and 518 nm, respectively. This type of particle was used to quantify the accumulation and distribution of MPs in mice. Another type was pristine PS particles, which was used for the toxicological test. In both cases we examined particles of two diameters (5 μm and 20 μm). 5 μm MPs was representative of the smaller size range of particles known to be ingested by many aquatic organisms^10^,^23^ and the smallest diameter of plastic debris found in marine habitats^14^. In addition, our previous study found that 5 μm diameter MPs could accumulated in the gills, liver and gut of zebrafish, while 20 μm diameter MPs could only accumulate in fish gills and gut^21^. The size of the particles was confirmed by scanning electron microscope (SEM) (Figure S1, Supporting Information). The composition of the particles was confirmed by FTIR spectroscopy (Figure S2). The aggregations of the particles detected by sedimentation experiment (Figure S3).

### Animal care

Five-week-old male mice (*Mus musculus*, ICR) were purchased from Qinglongshan Animal Breeding Center (Nanjing, China). The mice were housed in stainless-steel cages and acclimated for two weeks at 25 ± 4 °C, 50 ± 5% relative humidity, and a 12/12 h light/dark cycle. Food and water were provided *ad libitum*. All experimental processes were in accordance with NIH Guide for the Care and Use of Laboratory animals. The protocol was approved by the Committee on the Ethics of Animal Experiments of the Nanjing Military General Hospital.

### Tissue accumulation of MPs in mice

A total of 75 mice were randomly assigned to 15 cages (n = 5 for each cage). In one cage, mice were used as negative controls and treated with microplastics-free water. Seven cages of mice were treated with 5 μm fluorescent PS-MPs and 7 others were treated with 20 μm fluorescent PS-MPs. l mg MPs were dispersed in 5 mL Milipore Mili-Q water and treated by ultrasonic vibration, then 0.5 mL of the mixed solution was given once daily (0.1 mg/day) by oral gavage (1.46 × 10^6^ items for 5 μm PS-MPs and 2.27 × 10^4^ items for 20 μm PS-MPs). One cage of 5 mice from each of 5 μm and 20 μm MPs treatment groups were sacrificed at 1, 2, 4, 7, 14, 21, and 28 days after exposed to MPs. Tissue samples (liver, kidney and gut) were removed and frozen at −80 °C. In order to assess the retention of MPs in mice, another 10 mice were randomly assigned to 2 cages (n = 5 for each cage) and also exposed to the two sizes of fluorescent PS-MPs (0.1 mg/day by oral gavage) for 28 days. Then the exposure was terminated and one week later the mice were sacrificed and tissues samples were collected and stored at −80 °C.

After lyophilized, the dry tissues (0.1 g) were digested in nitric acid (1 mL, 65% V/V) at 70 °C for 2 h, and then digested in hydrogen peroxide (1 mL, 30% V/V) at 85 °C for 2 h. The digested solution was diluted with deionized water to a final volume of 10 mL. The concentrations of MPs in different tissues were determined by using a fluorescent spectrophotometer with excitation = 418 nm and emission = 518 nm. The standard curve was generated using serial dilutions of fluorescent PS-MPs suspensions (Figure S4). The background fluorescence of the tissues of control mice were detected and subtracted from that of MPs-treated samples. To confirm the accuracy of the standard curves, each assay was run in triplicate. The tissues were also fixed in 10% formalin, embedded in paraffin wax, sectioned at 4 μm thickness, and stained with hematoxylin and eosin (H&E) for final observation. In order to observe the presence of fluorescent-labeled polystyrene microplastic particles in tissues, one bright-field image was acquired by microscopy first and then a dark-field image of the same slide was acquired by epifluorescence microscopy. Finally, the two sets of images were stacked together by using AxioVision Rel. 4.7.

### Toxicological experiment

Acclimated mice were randomly assigned to control and PS-MPs treated groups (n = 5 for each group). Similar with above accumulation experiment, mice in treatment groups were exposed to 5 μm and 20 μm pristine PS-MPs with the exposure doses of 0.01 mg/day (1 × 10^5^ items for 5 μm PS-MPs and 2 × 10^3^ items for 20 μm PS-MPs), 0.1 mg/day (1 × 10^6^ items for 5 μm PS-MPs and 2 × 10^4^ items for 20 μm PS-MPs) and 0.5 mg/day (5 × 10^6^ items for 5 μm PS-MPs and 1 × 10^5^ items for 20 μm PS-MPs) by oral gavage. The low dose was selected based on previous toxicological studies of MPs in aquatic organisms^24^,^29^. The middle dose was chosen according to the environmentally realistic concentration of MPs in rivers (~10^6^ items m^−3^)^30^. The high dose was 5 times as much as the middle dose. All mice were sacrificed and examined after four weeks of exposure. Liver and serum samples were collected and stored at −80 °C.

### Histological analysis

The livers of mice from the group treated with 0.5 mg/day PS-MPs and the control group were fixed in 10% formalin, embedded in paraffin wax, sectioned at 4 μm thickness, and stained with H&E for microscopic observation.

### Biological analyses

To evaluate the toxic effects of PS-MPs, alterations of biomarkers in mice livers due to MPs exposure were determined. These markers included: energy metabolism in terms of ATP level and lactate dehydrogenase (LDH) activity; lipid metabolism as the level of total cholesterol (T-CHO) and triglycerides (TG); oxidative stress-related biomarkers, i.e. catalase (CAT), glutathione peroxidase (GSH-Px) and superoxide dismutase (SOD); neurotoxic responses in terms of acetylcholinesterase (AChE) activity. All these markers were detected by using commercial kit (Nanjing Jiancheng Bioeng. Inst., China), and the measurements were conducted according to the manufacture’s protocols.

### Metabolomic analysis

300 μL phosphate buffer (70 mM Na_2_HPO_4_; 0.025 (w/v) NaN_3_; 20% (v/v) D_2_O; 3 mM TSP; pH 7.4) was added into 300 μL serum. This mixture was homogenized and centrifuged at 12000 rpm for 10 min and then 550 μL of the supernatants were transferred into 5 mm NMR tubes. ^1^H-NMR spectra were acquired using a Bruker AV600 spectrometer (Bruker Co., Germany) at 298 K. The detailed detection methods and spectral processing have been described in previous study^31^. The metabolite resonances were identified according to Human Metabolome Database (HMDB) (www.hmdb.ca).

### Statistical analysis

Tissue accumulation of PS-MPs, biomarker responses and relative contents of serum metabolites in exposed mice were compared with control ones by one-way ANOVA and post-hoc comparison (Bonferroni) was used to discriminate between mean values. Level of significance was set at *P* < 0.05. These analyses were performed by SPSS 15 software (SPSS Inc., USA). For metabolomic data, multivariate principal component analysis (PCA) was used to explore the relationships among different treatments and cluster the samples into groups of homogeneous observations. The specific calculations were conducted using MetaboAnalyst 3.0.

The differential metabolites (DMs) between MPs treatment groups and control group were identified based on the following criteria: (i) *P* < 0.05, (ii) absolute value of fold change ≥1.2, and (iii) significant alteration in at least two dose groups. In order to visualize the variations of metabolites between treatment groups, a heat map was generated based on z-scores^32^. The z-scores of metabolites were calculated based on the following formula:


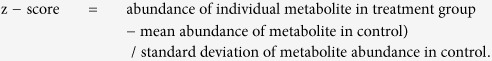


## Results

### Accumulation and distribution of MPs in mice tissues

**Figure 1:**
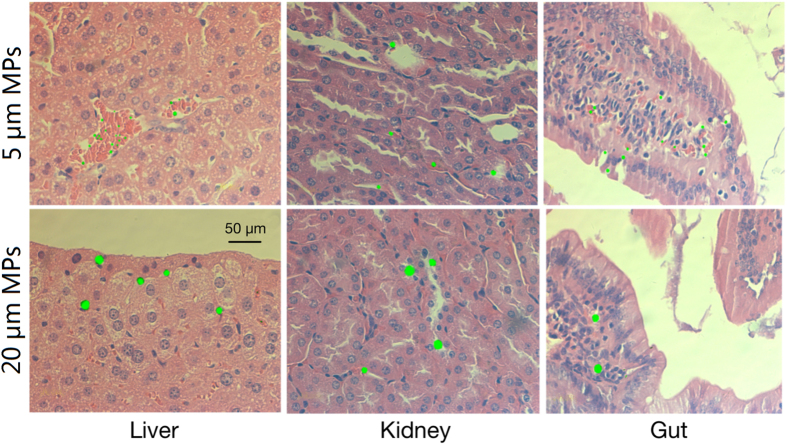
Accumulation of different sizes of MPs in mice tissues after exposure for 28 days.

**Figure 2:**
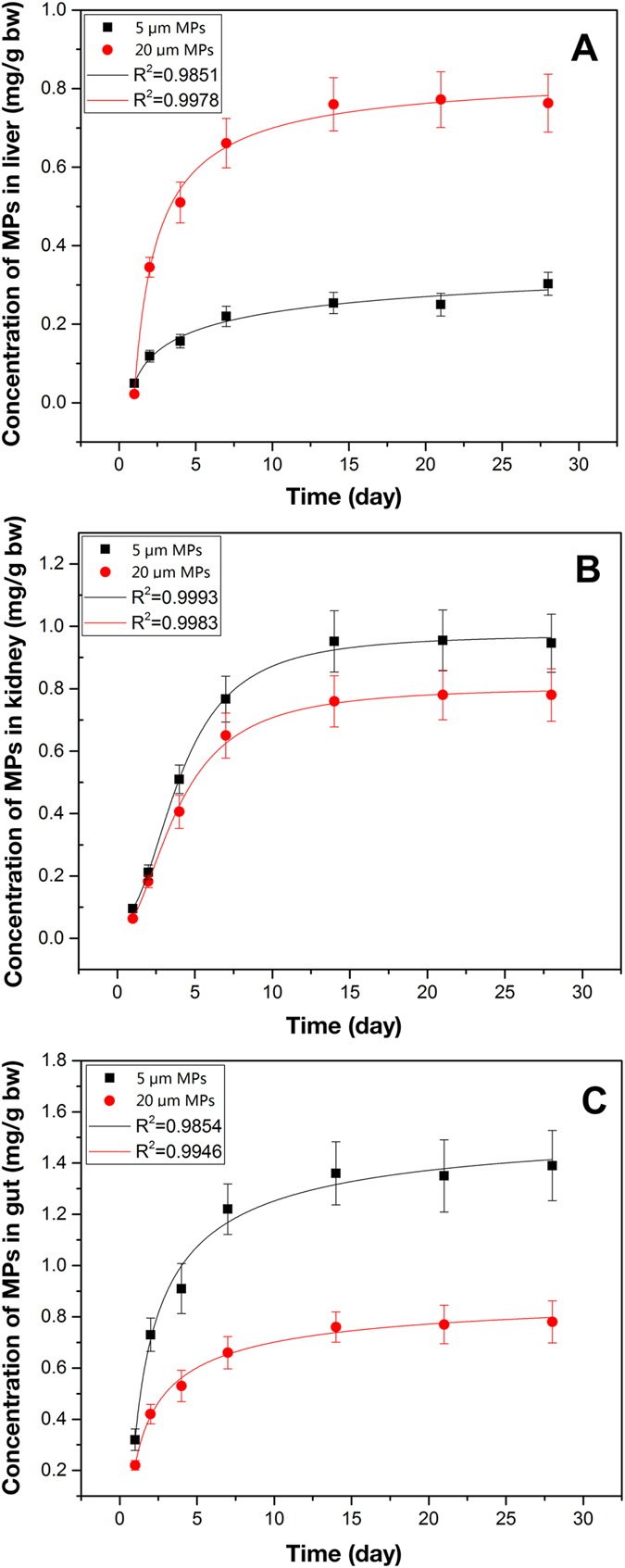
Concentration of MPs in 3 tissues of mice at different exposure times.

Both MPs sizes tested here displayed tissue accumulation over time (Fig. 1) and were clearly visible as distinct fluorescent points in the liver, kidney and gut of exposed mice. Throughout the experiment, the concentrations of PS-MPs in tissues were quantified using fluorescence spectroscopy based external standard calibration curves (Fig. 2). For both particle sizes tested, tissue concentration of MPs reached steady-state within 14 days of the onset of exposure in all three tissues (liver, kidney and gut). After 4 weeks of exposure, the maximal tissue concentrations of 5 μm MPs in the liver, kidney and gut were 0.303 ± 0.029 mg/g (4.42 × 10^6^ ± 4.23 × 10^5^ items/g), 0.946 ± 0.093 mg/g (1.38 × 10^7^ ± 1.36 × 10^6^ items/g), and 1.391 ± 0.137 mg/g (2.03 × 10^7^ ± 2.00 × 10^6^ items/g), respectively. For 20 μm MPs, the maximal concentrations accumulated in these three tissues were 0.763 ± 0.074 mg/g (1.73 × 10^5^ ± 1.68 × 10^4^ items/g), 0.783 ± 0.084 mg/g (1.78 × 10^5^ ± 1.91 × 10^4^ items/g), and 0.781 ± 0.082 mg/g (1.77 × 10^5^ ± 1.86 × 10^4^ items/g), respectively. The maximal concentrations of 5 μm MPs accumulated in kidney and gut were significantly higher than that of 20 μm MPs (*P* < 0.05). On the other hand, significantly fewer 5 μm MPs were retained in liver relative to 20 μm MPs after 4 weeks of exposure (*P* < 0.05). The two sizes of MPs could still be observed in the three tissues within one week after termination of the exposure (Figure S5).

### Adverse consequences of MPs in mice

All animals were alive after the 4 weeks of exposure. Full details about the body weight, organ weight, relative organ weight and food-intake are provided in Table S1. No significant changes for daily food consumption were found between control and MP treated groups. No significant differences were observed for the final body weight and liver weight between control and treatment groups. The relative weight of liver, however, significantly decreased in the high dose (0.5 mg/day) treatment groups. Among these groups, significantly increased food-intake was also observed in the mid and high dose treatment group of 20 μm MPs.

#### Histological lesions induced by MPs

**Figure 3:**
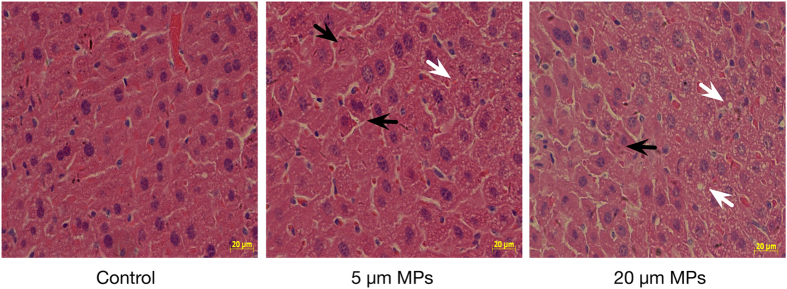
Representative images of H&E-stained liver sections from mice exposed for 4 weeks to 0.5 mg/day PS-MPs (5 μm and 20 μm). Black arrows indicate liver inflammation and white arrows indicate lipid droplets.

After the tissue accumulation experiments, 5 μm and 20 μm pristine PS-MPs were used for toxicological experiments. Representative histological sections of livers from mice exposed to 0.5 mg/day were examined (Fig. 3). Compared with control mice, inflammation and lipid droplets were observed in the livers of PS-MPs-treated mice.

#### Biological variations induced by MPs

**Figure 4:**
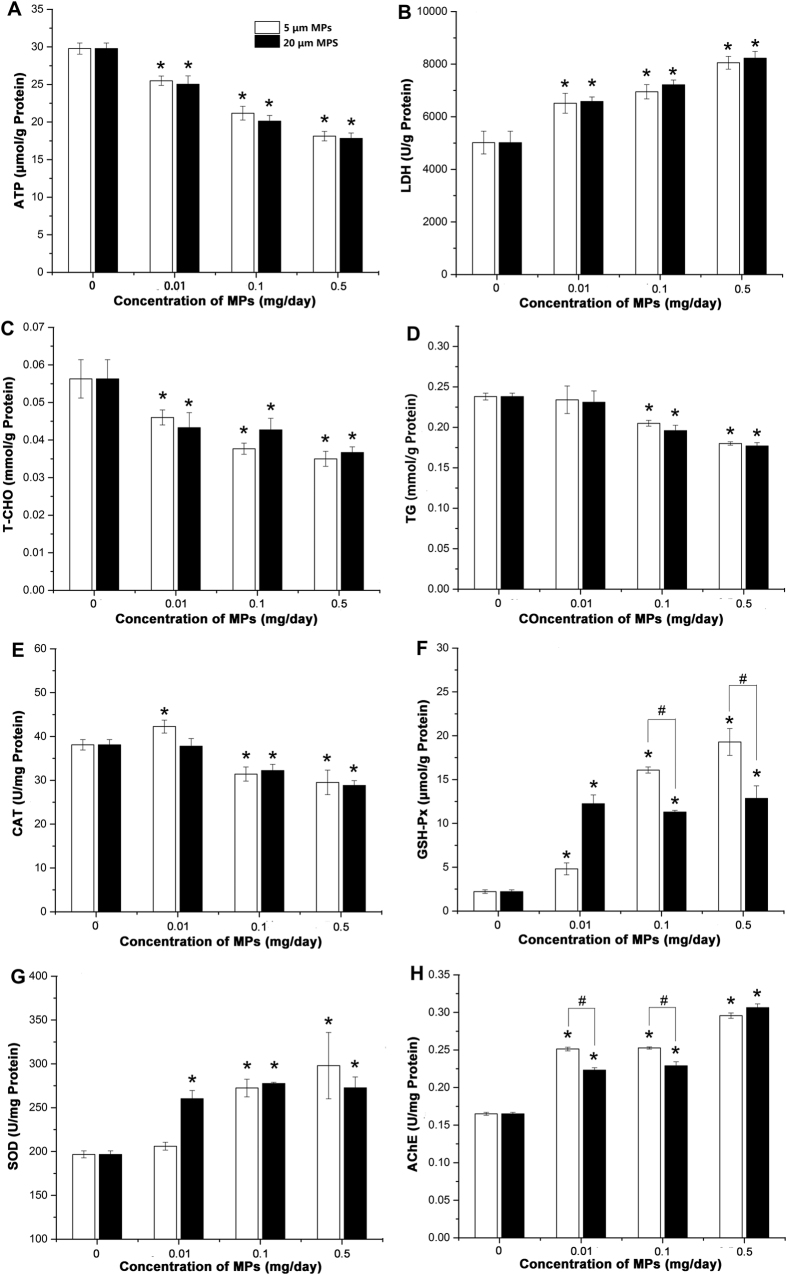
Effects of MPs exposure on biological markers related to energy metabolism, lipid metabolism, oxidative stress and neurotoxicity. (**A**) ATP levels; (**B**) LDH activities; (**C**) T-CHO levels; (**D**) TG levels; (**E**) CAT activities; (**F**) GSH-Px levels; (**G**) SOD activities and (**H**) AChE activities. *Means significant difference between MPs-treated groups and control group (*P* < 0.05). ^#^Means significant difference between different MPs-treated groups (*P* < 0.05).

Biological parameters related to energy metabolism, lipid metabolism, oxidative stress and neurotoxic responses were examined in livers (Fig. 4). Energy metabolism: both sizes of PS-MPs induced significant decrease in ATP level and significant increase in LDH activity in a dose-dependent manner (Fig. 4A). Lipid metabolism: we observed significant decreases in all treatments for the levels of T-CHO and TG (Fig. 4C and D). For biomarkers of oxidative stress: the activities of GSH-Px and SOD increased in the mice exposed to PS-MPs, while the activity of CAT decreased in almost all the treatment groups. In addition, potential for neurotoxicity was evaluated based on the activity of AChE in liver, which decreased after exposure to two sizes of PS-MPs. Noteworthy, most of these biological markers did not exhibit significant differences between 5 μm MPs treatment groups and 20 μm MPs treatment groups. On the other hand, the effects on GSH-Px and AChE in the 5 μm MPs treatment groups were greater than those in 20 μm MPs treatment groups.

#### Metabolomic alterations induced by MPs

**Figure 5:**
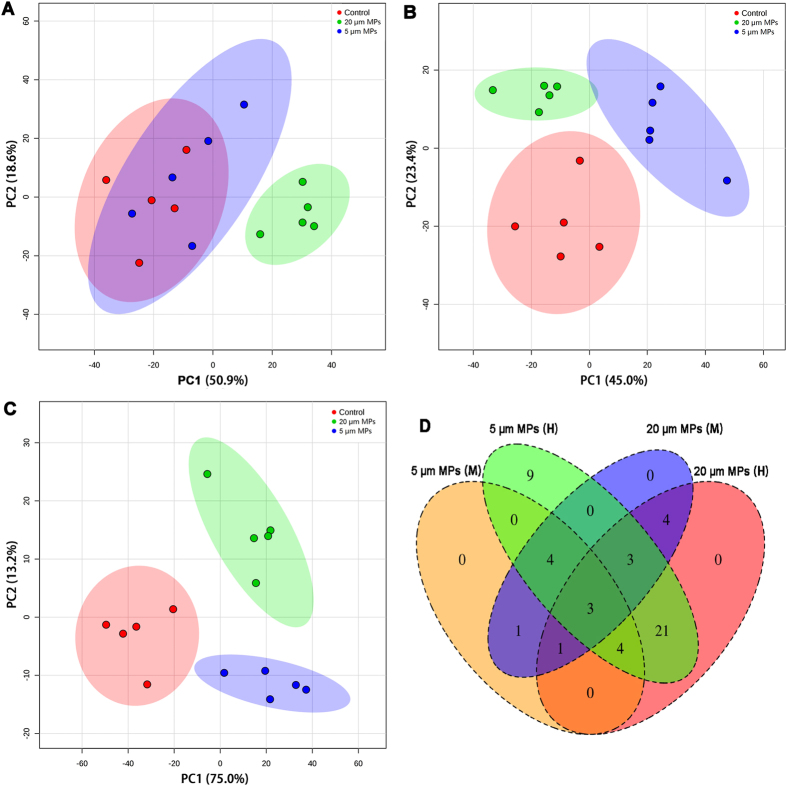
Metabolomic alterations due to MPs exposure. Scores plots of PLS-DA for low (**A**), mid (**B**) and high (**C**) doses of MPs-treated groups and control group. (**D**) Number of differential metabolites among MPs-treated groups. M, 0.1 mg/day; H, 0.5 mg/day.

Alterations in metabolic profiles of mice due to pristine PS-MPs exposure were determined. Representative ^1^H-NMR spectra of PS-MPs treated and control groups are provided in Figure S6, and a number of marker metabolites are identified (Table S2). Some apparent differences were observed between these spectra through visual comparison. To further visualize the treatment related differences, unsupervised PCA model was performed on these ^1^H-NMR data sets. For 5 μm MPs, mice exposed to low dose of MPs (0.01 mg/day) could not be separated from control mice based on the PCA score plots (Fig. 5A), while mice exposed to mid (0.1 mg/day) and high (0.5 mg/day) doses of MPs could be clearly separated from control mice (Fig. 5B and C). For 20 μm MPs, exposed mice could be clearly isolated from control and 5 μm MPs treated mice, no matter what doses were used (Fig. 5).

Differential metabolites (DMs, *P* < 0.05 and fold change ≥ 1.2 compared with control) in the mid and high dose treatments were further analyzed. A total of 50 DMs were found to be significantly different across the exposure groups. Among these DMs, 11 DMs were shared between mid and high doses of 5 μm MPs treated groups, 11 DMs were shared between mid and high doses of 20 μm MPs treated groups, and 3 DMs were shared among all four treatments (Fig. 5D).

**Figure 6:**
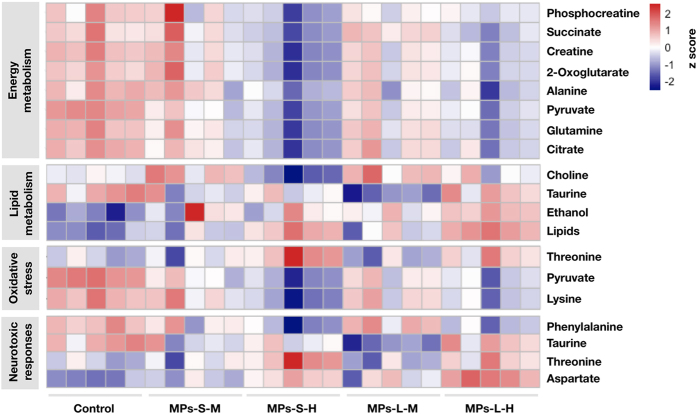
Heat map for the differential metabolites identified in different treatment groups calculated by z-scores. M, 0.1 mg/day; H, 0.5 mg/day.

Furthermore, 37 DMs were shared between 5 μm and 20 μm MPs treated groups and these metabolites involved in energy metabolism (8 DMs), lipid metabolism (4 DMs), response to oxidative stress (3 DMs), and response to neurotoxicity (4 DMs) (Fig. 6). In order to illustrate the variations of individual metabolite among the treatment groups, a heat map was generated based on *z*-scores of these DMs (Fig. 5). Phosphocreatine, succinate, creatine, 2-oxoglutarate, alanine, pyruvate, glutamine, citrate, choline, lysine and phenylalanine significantly decreased, while taurine, threonine, lipids and aspartate significantly increased with increasing doses of MPs.

## Discussion

High concentrations of MPs have been detected in the oceans (0–1 × 10^4^ items/m^3^)^33^ and fresh waters (0–1 × 10^6^ items/m^3^)^30^. MPs have also been observed in various species of animals, especially mussels and fish used for human consumption. It is now evident that MPs can be transmitted through the aquatic food chain, which is expected to lead to its biological accumulation. For instance, it was found that mussels accumulated MPs up to concentration of 2 items/g tissues in contaminated environment^34^. MPs were also detected in fish from the English Channel (1.90 ± 0.10 items per fish)^15^. Therefore, food products may represent an important route of entry for MPs into humans, although systematic quantitative data of MPs in human tissues have not been reported. Furthermore, several studies have observed MPs in some non-marine food products, such as honey (40–660 items/kg honey), sugars (32 ± 7 items/kg sugar), beer (12–109 items/L beer) and table salt (7–681 items/kg salts)^35^. These represent additional dietary routes for human populations to be exposed to MPs. However, there is not published data on human tissues exposed to MPs from any source and it remains unclear if MPs would accumulate in mammalian tissues. Using fluorescent MPs, fluorescence spectroscopy and histological analyses we traced the accumulation and distribution of MPs of two sizes in the livers, kidneys and guts of mice. We observed MPs of both sizes in all three tissues. Collectively, the data support the hypothesis that MPs not only accumulate in digestive system but that they are also transported to other tissues through the circulatory system.

The data indicated that the distribution of MPs in tissues is partially determined by particle size. Interestingly, the accumulations of large particles (20 μm diameter) appeared consistently distributed among all tissues, whereas small particles (5 μm diameter) displayed higher accumulation in the gut. Different patterns of distribution of MPs in tissues have also been demonstrated in aquatic organisms. For example, 2–16 μm MPs mainly accumulated in the gut cavity and digestive tubules of M. *edulis* via oral ingestion^20^. MPs with sizes of 8–10 μm largely accumulated in crabs gill via the ventilation and in crabs gut through oral ingestion^14^. On the other hand, 5 μm MPs accumulated in gut and kidney to a much markedly higher concentration than that achieved by 20 μm MPs. Although mechanisms still remain to be elucidated, our results and those of others support the hypothesis that small particle size enhances the accumulation of MPs in tissues^36^,^37^,^38^. Similarly, previous studies demonstrated that orally administrated nanoparticles could distribute in various tissues in mice and the amount of accumulation and distribution inversely correlated with the particle sizes^39^,^40^.

Most research on the toxicity of MPs has focused on their impacts on aquatic organisms in contaminated environments. However, few studies have evaluated the adverse effects of MPs on mammals. In this study, a battery of biomarkers and metabolomic profiles were used to characterize the potential toxic effects of MPs in mice. The documented effects highlight impacts on energy metabolism, lipid metabolism, oxidative stress, and neurotoxic responses.

### Energy metabolism

Impairment of energy metabolism was observed in MPs exposed mice with a significant reduction in ATP concentration and a sharp increase in LDH activity in liver. ATP level and LDH activity in liver are related to the energy content of the liver^41^. In agreement with these observations we also detected serum metabolites known to be involved in energy metabolism, such as creatine, 2-oxoglutarate, and citrate^42^. These results suggest that MPs exposure resulted in energy deficiency in mice, which can also be confirmed by the decreased relative weight of liver and increased food-intake in high dose of MPs treatment groups. The findings are also in agreement with reports that MPs could deplete energy reserves in marine worms and copepods^43^. Ingested MPs could affect the normal absorption of food and inhibit food digestion.

### Lipid metabolism

The accumulation of MPs in tissues caused a significant decrease of T-CHO and TG, suggesting that disturbance of lipid metabolism was induced by the exposure of MPs particles. Additionally, metabolites involved in lipid metabolism^42^,^43^ such as taurine, ethanol and a variety of lipids increased while choline decreased in the serum of MPs-treated mice. Interestingly, histological analyses revealed the accumulation of lipid droplets in the livers of MPs-treated mice. Lipid droplets are highly dynamic and play an important role in the regulation of intracellular lipid storage and lipid metabolism. Lipid droplets in hepatocytes are frequently observed phenotype in inflammatory conditions and are used as a marker for inflammatory responses^44^. Similarly, disturbed lipid metabolism and lipids accumulation was also observed in mussels and fish exposed to MPs^16^,^45^. The possible reason is that MPs exposure may cause inflammatory responses, which has been observed in the MPs exposed groups, and thus lead to lipid disturbance in the livers^46^.

### Oxidative stress

Exposure to MPs also induced responses to oxidative stress pathways in mice with increased GSH-Px and SOD and decreased CAT. These are sensitive markers to evaluate the early oxidative damages of environmental chemicals. These enzymes are all responsible for eliminating various reactive oxygen species (ROS)^31^. Meanwhile, substantial alterations were also observed in metabolites related to oxidative stress. For example, pyruvate and lysine decreased and threonine increased due to MPs exposure. These metabolites mainly act as endogenous antioxidants and protect against the toxicities of various xenobiotics^47^,^48^. These results indicated that MPs exposure could induce imbalance in the antioxidant defense system in mice.

### Neurotoxic responses

The activity of AChE in liver is one of the most common biomarkers of neurotoxicity^49^. Previous studies have been demonstrated that MPs exposure could affect AChE in aquatic organisms^25^,^45^. Here we observed that MPs treatments increased the activity of AChE, which may lead to a reduction of cholinergic neurotransmission efficiency^50^. Corresponding to a potentially neurotoxic response, MPs exposure also caused increases of threonine, aspartate and taurine in serum. All these metabolites act as neurotransmitter substances. In addition, phenylalanine, a precursor of neurotransmitters, was decreased. These alterations raise the possibility that MPs exposure could induce adverse effects on neurotransmission in mice.

**Figure 7:**
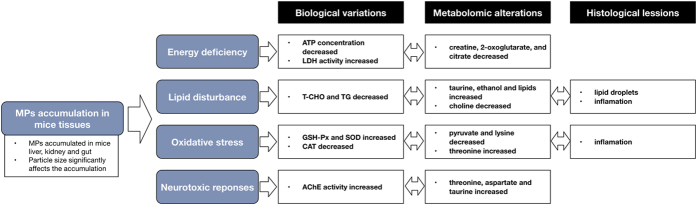
Summary of findings.

In conclusion, our results confirmed that MPs can accumulate in at least 3 tissues of mice (liver, kidney and gut) in a manner that depends on the particle size. Additionally, MPs accumulation induced several effects on biochemical biomarkers and metabolomic profiles highlighting the potential health risk to mammals (Fig. 7). Based on a comprehensive analysis of multiple indicators, we find evidence that MPs exposure could cause disruptions to energy and lipid metabolism, induce oxidative stress, and include neurotoxic responses.

## Additional Information

**How to cite this article:** Deng, Y. *et al*. Tissue accumulation of microplastics in mice and biomarker responses suggest widespread health risks of exposure. *Sci. Rep.* **7**, 46687; doi: 10.1038/srep46687 (2017).

**Publisher's note:** Springer Nature remains neutral with regard to jurisdictional claims in published maps and institutional affiliations.

## Supplementary Material

Supplementary Information
