# Supplementary material for: Tissue accumulation of microplastics in mice and biomarker responses suggest widespread health risks of exposure
Source: Sci Rep. 2017 Apr 24;7:46687. doi: 10.1038/srep46687 (PMC5402289; doi:10.1038/srep46687)
Supplement: Supplementary Information [file srep46687-s1.docx]

**Supporting Information**

Tissue accumulate of microplastics in mice and biomarker responses suggest widespread health risks of exposure

Yongfeng Deng^1^, Yan Zhang*^1^, Bernardo Lemos^2^, Hongqiang Ren^1^

1. State Key Laboratory of Pollution Control and Resource Reuse, School of the Environment, Nanjing University, Nanjing, Jiangsu 210023, China
2. Program in Molecular and Integrative Physiological Sciences, Department of Environmental Health, Harvard T. H. Chan School of Public Health, Boston, MA 02115, USA

**Corresponding author:**

Yan Zhang ([yanzhang@nju.edu.cn)](mailto:yanzhang@nju.edu.cn))

This Supporting Information contains:

| **Page S2:** | The images for MPs detected by scanning electron microscope and fluorescence microscopy, **Figure S1** |
| --- | --- |
| **Page S3:** | FTIR spectroscopy for 5μm and 20 μm PS-MPs, **Figure S2** |
| **Page S4:** | The aggregation of 5 μm and 20 μm PS-MPs was determined by fluorescence spectrometry, **Figure S3** |
| **Page S5:** | The standard cures used for quantitatively detection of MPs in **t**issues, **Figure S4** |
| **Page S6:** | Retention of two sizes of MPs in mice tissues within one week after termination of the exposure, **Figure S5** |
| **Page S7:** | Representative ^1^H-NMR spectra of serum samples, **Figure S6** |
| **Page S8:** | Bodyweight, liver weight, relative liver weight and food intake of mice after four weeks of treatment with MPs, **Table S1** |
| **Page S9:** | Metabolites assignment of NMR spectra for serum and urine samples, **Table S2** |

**Figure S1.** The images for MPs detected by scanning electron microscope (SEM) and fluorescence microscopy. (A) 5 μm fluorescent PS-MPs. (B) 5 μm pristine PS-MPs. (C) 20 μm fluorescent PS-MPs. (D) 20 μm pristine PS-MPs.


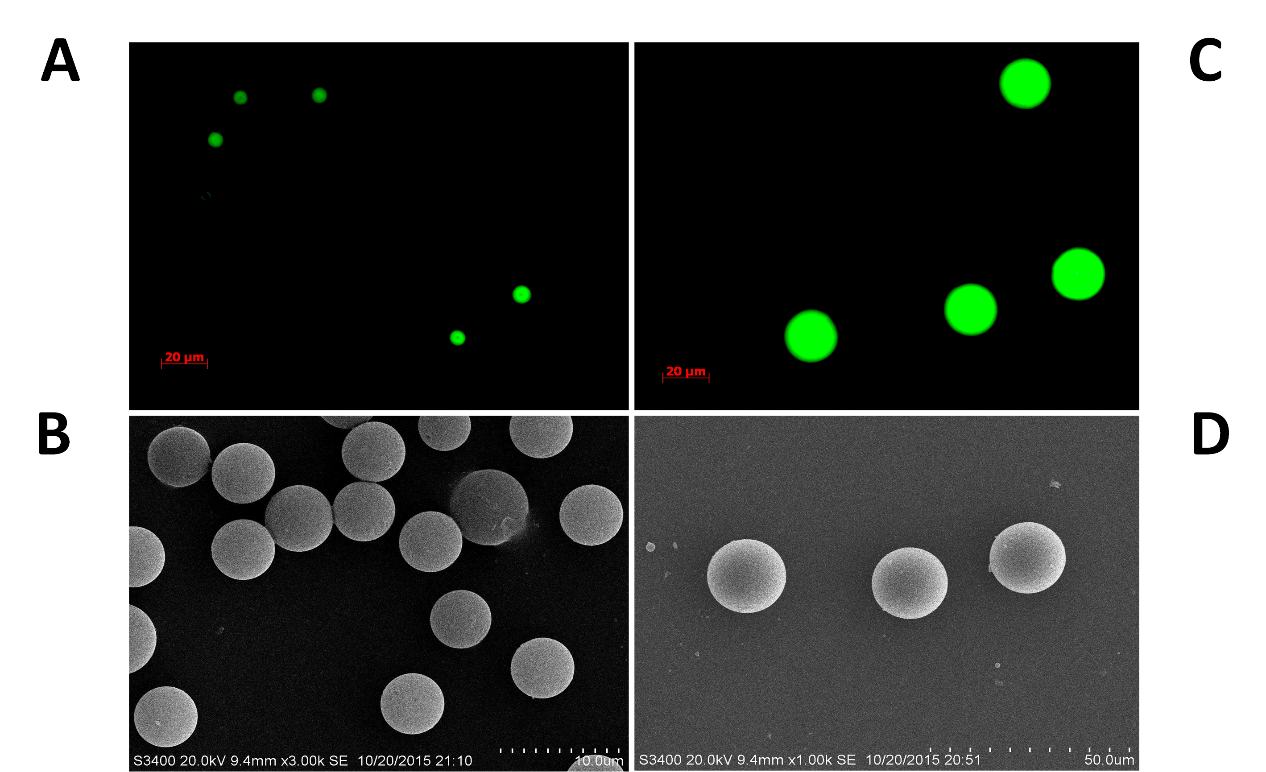


**Figure S2.** FTIR spectroscopy for 5 μm PS-MPs (A) and 20 μm PS-MPs (B).


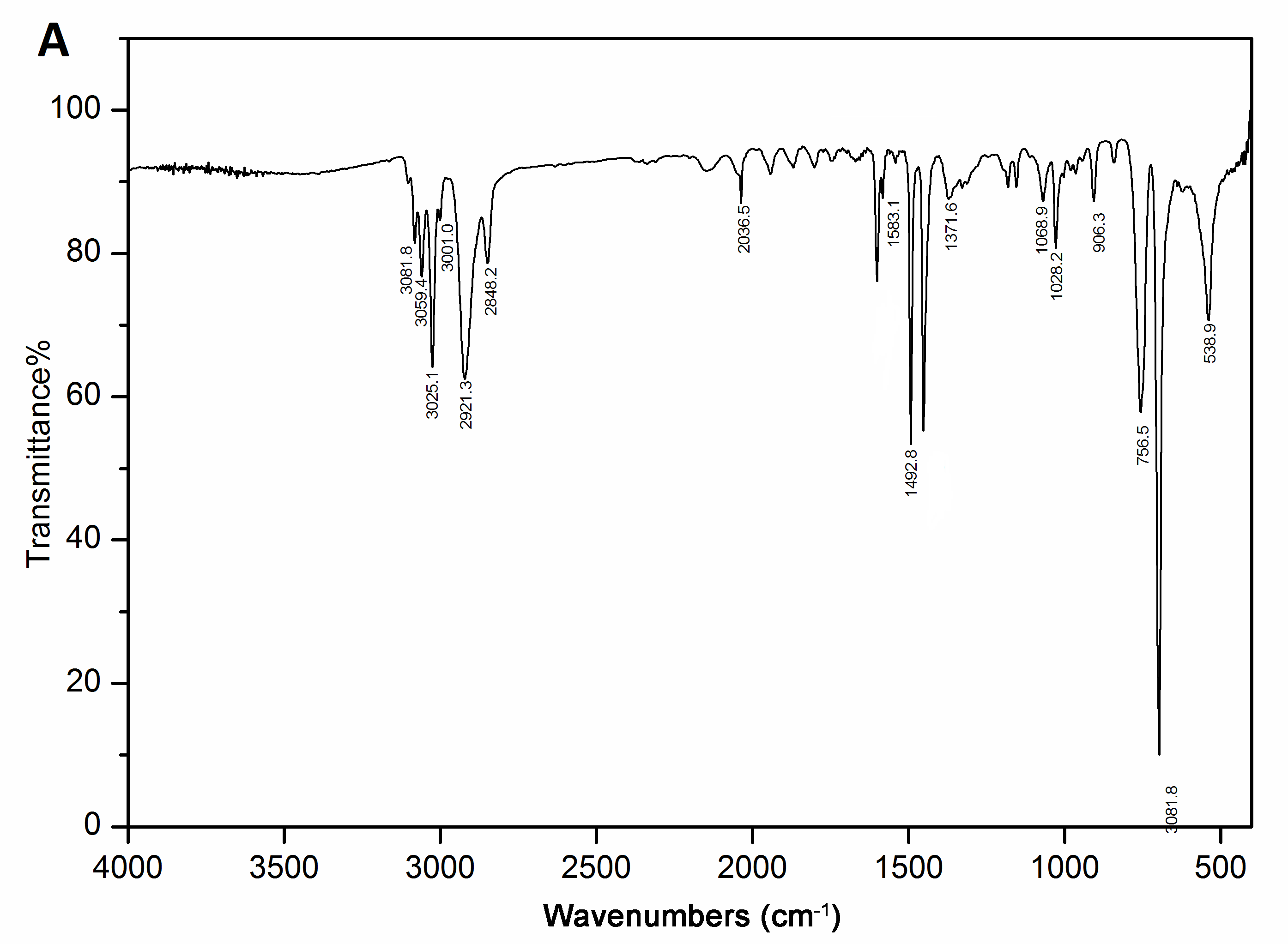


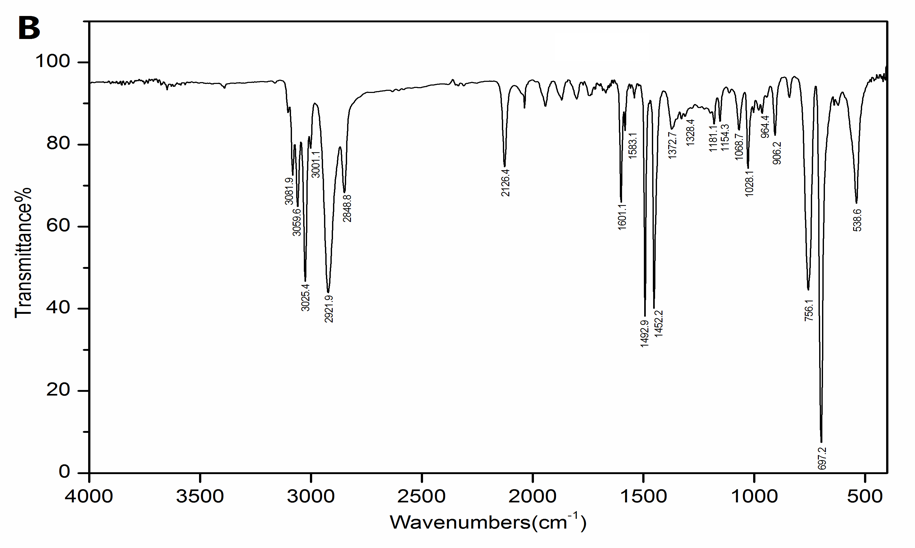


**Figure S3.** The aggregation of 5 μm and 20 μm PS-MPs was determined by fluorescence spectrometry.


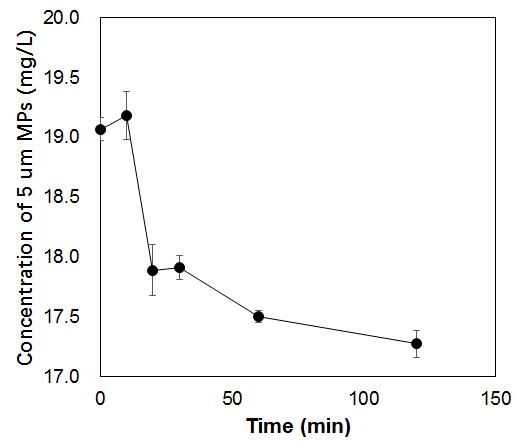

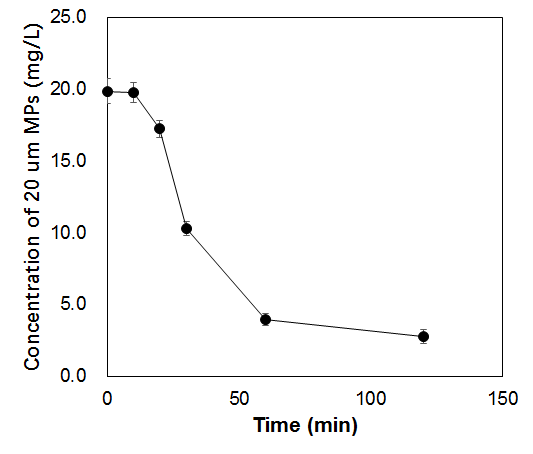


*Methods:*

To determine the aggregation, 5 and 20 μm fluorescent particles (20 mg/L) were respectively added into four tanks (containing 500 mL test solution and two tanks for each size of particle). After sonicated for 10 min, two tanks were aerated and other two tanks were allowed to stand, and then a 3 mL sample was taken from the center of the tank at 10 min, 20 min, 30 min, 1 h and 2 h. The concentration of the fluorescent PS-MPs was determined by using a standard curve generated by measuring the fluorescence of known concentrations of MPs on a fluorescence spectrophotometer (HITACHI F-7000; excitation = 418 nm, emission = 518 nm).

**Figure S4.** The standard curves used for quantitatively detection of MPs in **t**issues.


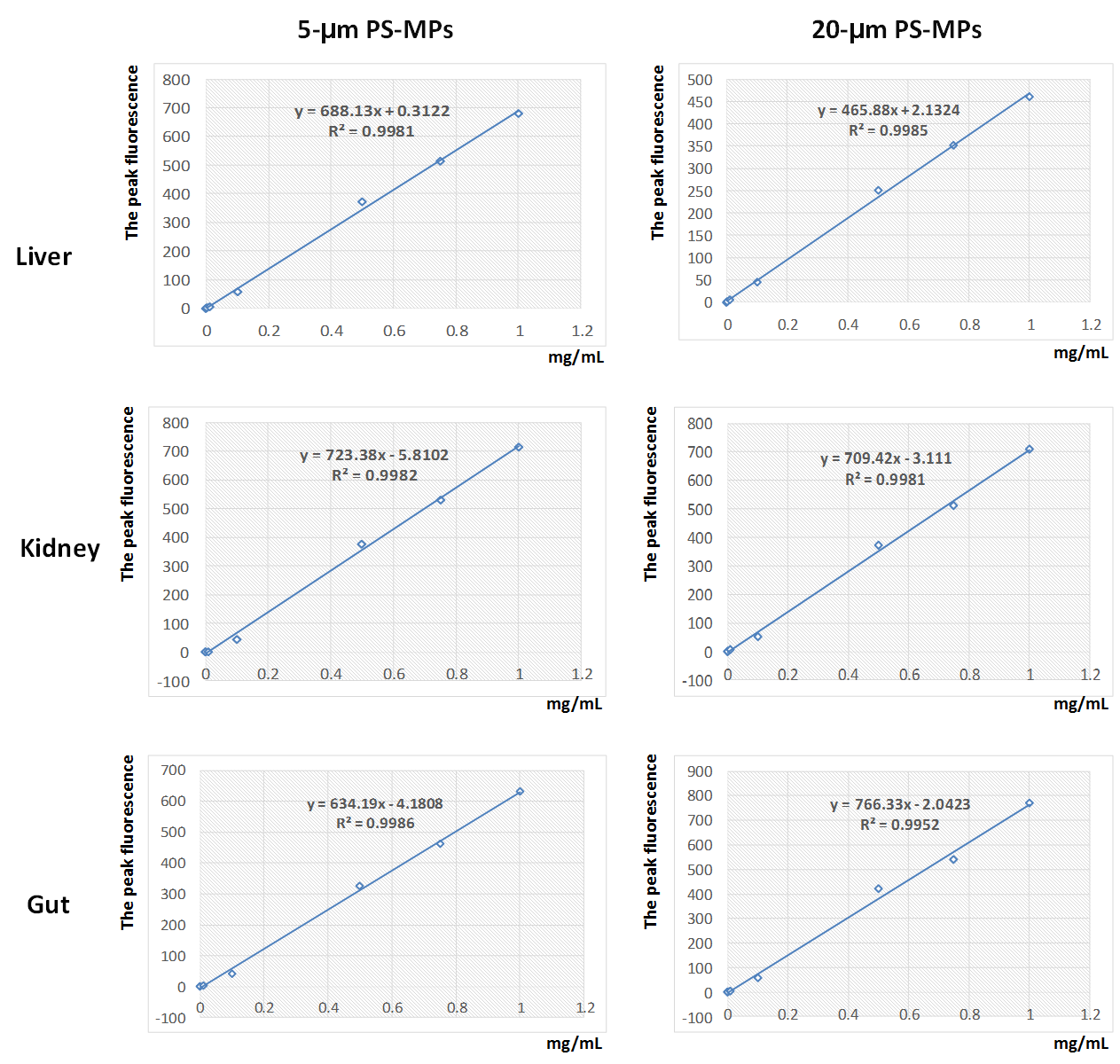


**Figure S5.** Retention of two sizes of MPs in mice tissues within one week after termination of the exposure.


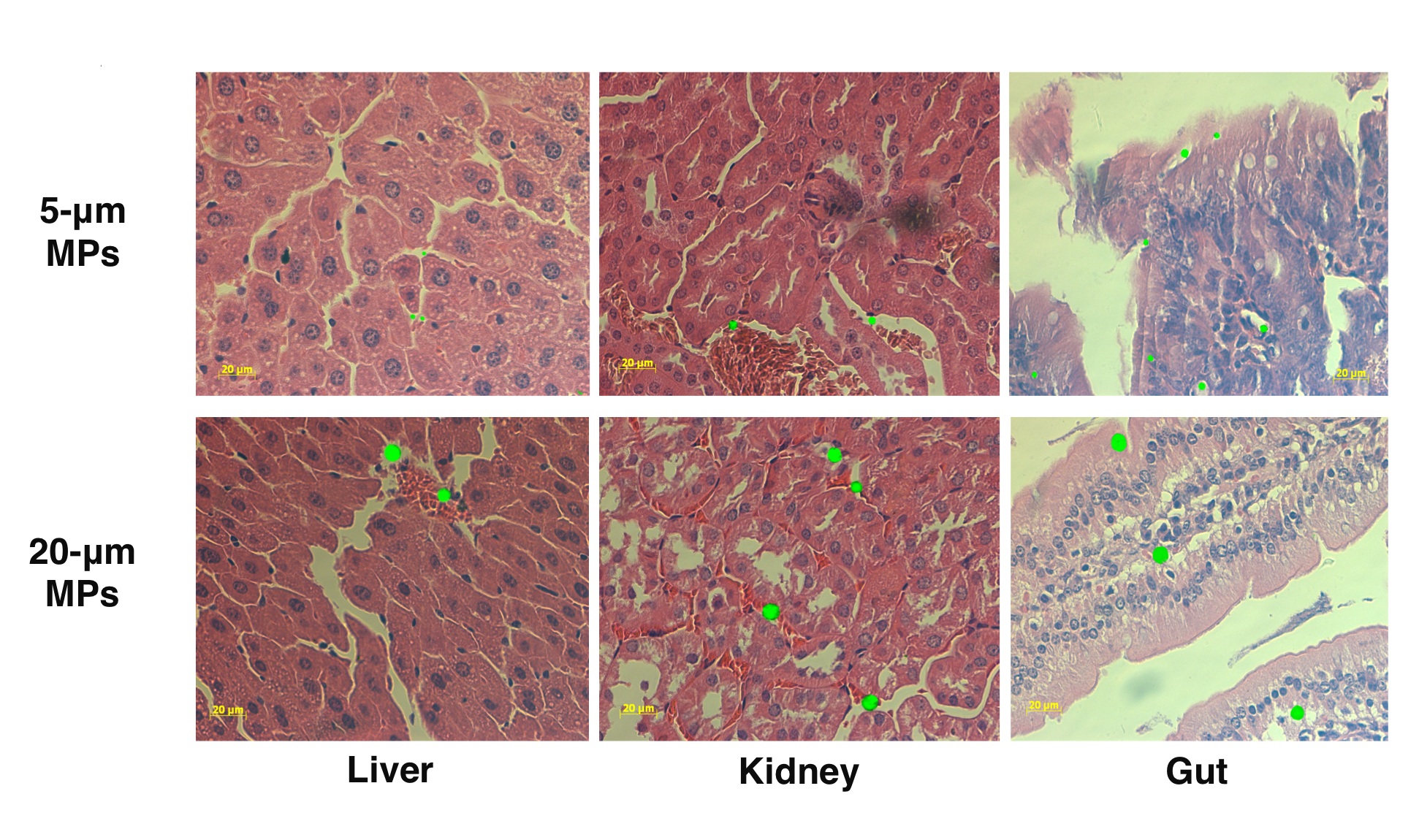


**Figure S6.** Representative ^1^H-NMR spectra of serum samples from (A) control group, (B) treatment group exposed to 0.1 mg/d 5 μm PS-MPs, (C) treatment group exposed to 0.5 mg/d 5 μm PS-MPs, (D) 0.1 mg/d 20 μm PS-MPs and (E) 0.5 mg/d 20 μm PS-MPs. Key to numbering in Table S2.


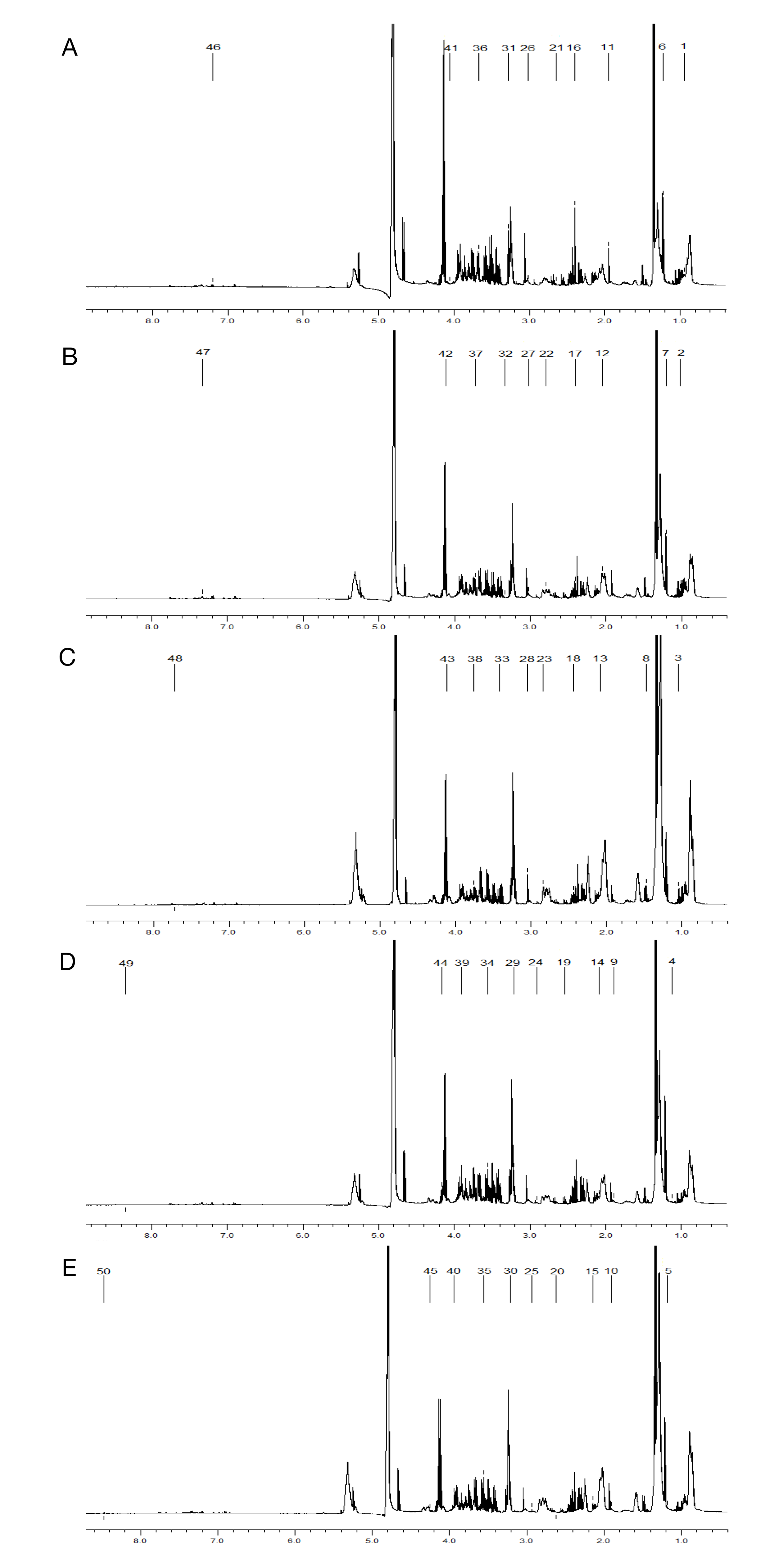


**Table S1.** Bodyweight, liver weight, relative liver weight and food intake of mice after four weeks of treatment with MPs (* *p* < 0.05).

|  | Control | 5 μm MPs | | | 20 μm MPs | | |
| --- | --- | --- | --- | --- | --- | --- | --- |
|  |  | 0.01 mg/day | 0.1 mg/day | 0.5 mg/day | 0.01 mg/day | 0.1 mg/day | 0.5 mg/day |
| Initial bodyweight (g) | 36.30 ± 1.23 | 35.74 ± 1.42 | 36.10 ± 1.16 | 37.01 ± 1.31 | 35.84 ± 1.63 | 36.14 ± 1.73 | 36.15 ± 1.97 |
| Final bodyweight (g) | 40.47 ± 1.38 | 37.83 ± 4.30 | 40.43 ± 2.47 | 36.77 ± 4.04 | 35.90 ± 0.93* | 37.89 ± 2.32 | 40.43 ± 0.55 |
| Liver weight (g) | 1.74 ± 0.13 | 1.55 ± 0.25 | 1.73 ± 0.03 | 1.47 ± 0.33 | 1.57 ± 0.23 | 1.55 ± 0.33 | 1.56 ± 0.34 |
| Relative liver weight | 0.043 ± 0.003 | 0.041 ± 0.002 | 0.043 ± 0.002 | 0.038 ± 0.001* | 0.044 ± 0.003 | 0.041 ± 0.002 | 0.039 ± 0.002* |
| Food intake (g) | 38.63 ± 4.40 | 36.21 ± 3.60 | 37.62 ± 5.86 | 38.48 ± 4.21 | 36.80 ± 4.83 | 45.16 ± 4.91* | 44.73 ± 4.27* |

**Table S2.** Metabolites assignment of NMR spectra for serum and urine samples.

| No. | Metabolite | Chemical Shift (ppm) | Moieties |
| --- | --- | --- | --- |
| 1 | Leucine | 0.94 (d) | δCH_3_ |
| 2 | Isoleucine | 1.02 (d) | δCH_3_ |
| 3 | Valine | 1.05 (d) | γCH_3_ |
| 4 | α-Ketoisovalerate | 1.13 (d) | CH_3_ |
| 5 | Ethanol | 1.18 (t) | βCH_3_ |
| 6 | 3-D-hydroxybutyrate | 1.20 (d) | γCH_3_ |
| 7 | 3-Hydroxy-isovalerate | 1.21 (s) | CH_3_ |
| 8 | Alanine | 1.47 (d) | βCH_3_ |
| 9 | Citrulline | 1.88 (m) | CH_2_ |
| 10 | Arginine | 1.89 (m) | βCH_2_ |
| 11 | Acetate | 1.92 (s) | CH_3_ |
| 12 | N-acetyl-glycoproteins (Nac) | 2.04 (s) | CH_3_ |
| 13 | O-acetyl-glycoproteins | 2.07 (s) | CH_3_ |
| 14 | Glutamate | 2.08 (m) | βCH_2_ |
| 15 | Methionine | 2.14 (s) | δCH_3_ |
| 16 | Succinate | 2.39 (s) | CH |
| 17 | Pyruvate | 2.41 (s) | CH_3_ |
| 18 | Glutamine | 2.44 (m) | γCH_2_ |
| 19 | Glutathione | 2.55 (m) | CH_2_ |
| 20 | α-Keto-isocaproate | 2.61 (d) | CH_2_ |
| 21 | Citrate | 2.65 (d) | CH_2_ |
| 22 | Lipids | 2.78 (m) | =C-CH_2_-C= |
| 23 | Aspartic acid | 2.82 (m) | βCH_2_ |
| 24 | Trimethylamine(TMA) | 2.91 (s) | CH_3_ |
| 25 | Dimethylglycine (DMG) | 2.93 (s) | CH_2_ |
| 26 | 2-Oxoglutarate | 3.01 (t) | CH_2_ |
| 27 | Isobutyrate | 3.02 (m) | CH |
| 28 | Creatine | 3.04 (s) | N-CH_3_ |
| 29 | Choline | 3.21 (s) | N-(CH_3_)_3_ |
| 30 | Phosphorylcholine | 3.23 (s) | N(CH_3_)_3_ |
| 31 | Trimethylamine-N-oxide (TMAO) | 3.27 (s) | CH_3_ |
| 32 | Scyllo-inositol | 3.33 (s) | OH |
| 33 | Taurine | 3.41 (t) | S-CH_2_ |
| 34 | Glycerol | 3.56 (m) | CH_2_ |
| 35 | Glycine | 3.56 (s) | CH_2_ |
| 36 | Phenylacetyl-glycine(PAG) | 3.68 (s) | 10-CH |
| 37 | Indoleacetyl-glycine(IAG) | 3.73 (s) | CH_2_ |
| 38 | Lysine | 3.76 (t) | αCH |
| 39 | Asparagine | 3.90 (m) | αCH |
| 40 | Phosphocreatine | 3.95 (s) | CH_2_ |
| 41 | Creatinine | 4.06 (s) | CH_2_ |
| 42 | Lactate | 4.11 (q) | αCH |
| 43 | Proline | 4.12 (t) | αCH |
| 44 | Kynurenine | 4.16 (t) | CH |
| 45 | Threonine | 4.25 (m) | βCH_2_ |
| 46 | Tyrosine | 7.17 (m) | CH |
| 47 | Phenylalanine | 7.33 (m) | 3,5-CH |
| 48 | Tryptamine | 7.70 (d) | 4-CH |
| 49 | Inosine | 8.34 (s) | 2-CH |
| 50 | Formate | 8.46 (s) | CH |
